# Sucrose interferes with endogenous cytokinin homeostasis and expression of organogenesis-related genes during de novo shoot organogenesis in kohlrabi

**DOI:** 10.1038/s41598-021-85932-w

**Published:** 2021-03-22

**Authors:** Tatjana Ćosić, Václav Motyka, Jelena Savić, Martin Raspor, Marija Marković, Petre I. Dobrev, Slavica Ninković

**Affiliations:** 1 Institute for Biological Research “Siniša Stanković”-National Institute of Republic of Serbia, University of Belgrade, Bulevar despota Stefana 142, 11060 Belgrade, Serbia; 2 Institute of Experimental Botany of the Czech Academy of Sciences, Rozvojová 263, 16502 Prague 6, Czech Republic

**Keywords:** Plant sciences, Plant development, Plant physiology, Genetics, Gene expression, Gene regulation

## Abstract

Cross-talk between phytohormones and sugars is intensely involved in plant metabolism, growth and regeneration. We documented alterations in cytokinin (CK) homeostasis in four developmental stages during de novo shoot organogenesis (DNSO) of kohlrabi (*Brassica oleracea* var. *gongylodes* cv. Vienna Purple) seedlings induced by exogenous CKs, *trans*-zeatin (*trans*Z) and thidiazuron (TDZ), added together with elevated sucrose concentration (6% and 9%). Significant impact of CK and sucrose treatment and their interaction was recorded in all investigated stages, including plantlet development before calli formation (T1 and T2), calli formation (T3) and shoot regeneration (T4). Results showed remarkable increase in total CK levels for *trans*Z treatment, particularly with 9% sucrose. This trend was observed for all physiological and structural groups of CKs. Application of TDZ contributed to little or no increase in CK levels regardless of sucrose concentration. Analysis of expression profiles of organogenesis-related genes involved in auxin transport, CK response, shoot apical meristem formation and cell division revealed that higher sugar concentration significantly downregulated the analysed genes, particularly in T3. This continued on TDZ, but *trans*Z induced an opposite effect with 9% sucrose in T4, increasing gene activity. Our results demonstrated that phytohormone metabolism might be triggered by sucrose signalling in kohlrabi DNSO.

## Introduction

Cytokinins (CKs) function as important metabolites essential for the regulation of plant development by affecting apical dominance, leaf morphology and senescence, cell propagation, root and shoot growth, embryogenesis, nutritional signalling and source-sink relations^1–3^. Exogenous plant growth regulators (PGRs), particularly CKs, can show variations in influencing de novo shoot organogenesis (DNSO) due to different uptake from the growth media and also the competence of the given genotype or explant type to metabolise these hormones^4^.

Furthermore, CK homeostasis of plants is much dependent on the presence of exogenous CKs in growth media^5,6^, which are also shown to be involved in upregulating auxin biosynthesis and transport, leading to elevation of endogenous auxin levels^6–8^. These effects could be achieved through regulating activity of auxin efflux transporters, such as PIN (PIN-FORMED) family^9^. Moreover, specific negative regulators of the CK biosynthetic pathway, A-type ARR (ARABIDOPSIS RESPONSE REGULATOR) proteins can influence the levels of CKs in plant tissues and thereby affect various steps of the CK signalling pathway^10,11^. One of the further elements of the CK cascade that was shown to be affected by altered CK homeostasis during DNSO due to the presence of exogenous CKs in the growth medium is the gene *RGD3* (*ROOT GROWTH DEFECTIVE3*)^12^, whose protein product is involved in controlling the development of adventitious shoot apical meristem (SAM)^13^. Underlying the process of SAM formation are coordinated cell divisions that have previously been shown to be under the regulation of phytohormones, particularly auxin and cytokinin^12,14,15^. CKs affect the phase-specific binding of cyclins with distinctive cyclin-dependent kinases (CDKs) forming the specific complex^16^ and regulate the transition from G2 to M phase^17,18^, thus controlling the cell cycle.

Sugars have been shown to function as signalling molecules whose transduction pathways affect metabolic and developmental processes in plants, potentially interacting with hormonal regulation^19–21^ and sustaining tight control on transcriptional, posttranscriptional and posttranslational levels^22,23^. Previous studies suggest that sugars can influence pathways activated by phytohormones through altering expression and activity of components of those pathways^20^.

The accessibility of sugars or their deficit initiates various metabolic and developmental responses in plants, so it is expected that sugars intensely affect the expression of many genes^24^. Different genes demonstrate diverse responses to specific sugar actions illustrating the existence of more than one signal transduction pathway^19,24,25^. Various studies revealed sugar-responsive expression of genes involved in the process of photosynthesis^22^. Sucrose-specific induction of gene expression for different promoters, such as patatin^26^ and phloem-specific rolC promoter^27^ was also demonstrated. Since then, a number of papers were published validating the impact of sucrose and glucose on gene expression^24^. For example, sucrose as well as glucose was reported to influence the activity of MYB proteins from rice, involved in gibberellin response^28^. Glucose also induced the activity of certain genes involved in abscisic acid (ABA) biosynthesis^29^. Besides PGRs, sucrose has been demonstrated to associate with regulation of cyclin activity affecting cell division^15,30^. Moreover, a great number of sugar-responsive transcription factors from the ERF/AP2, WRKY and bZIP families have been identified as crucial components of sugar-regulated gene expression^24^.

Sugar sensing and signalling have been intensively explored due to the significance of sugars in each aspect of plant growth and development. They occur at the level of a single cell, but the responses must be further assimilated to the tissues, organs, and ultimately the whole plant, indicating the need for interaction of those sugar-induced signals with other signalling pathways^25^. Studies have shown the existence of three diverse sugar-sensing systems: (1) hexokinase (HXK)-sensing system, (2) hexose transport-associated sensor, and (3) Suc-specific pathway, which may implicate a signalling Suc transporter^25^. Germination, hypocotyl elongation, flowering, senescence, root and leaf growth, metabolism of carbon and nitrogen, pathogen attack and wounding are some of the processes that were shown to be influenced by glucose signalling via HXK-dependent pathways^31^.

Plant growth regulators like ABA, gibberellins and CKs are implicated in regulating processes associated with sugar metabolism and transport^20^. Predominantly, CKs have earlier been proposed to be involved in mediating source-sink relations, and there are numerous examples indicating an existence of CKs and sugar interactions that may affect their signalling pathways^19,32–34^. For example, sucrose was shown to downregulate the expression of the group-3 SNF1-related protein kinase WPK4 from wheat, which is initiated by CKs^35^. Furthermore, besides a strong effect of glucose on the genes involved in CK metabolism and signalling, glucose and CKs were demonstrated to act agonistically as well as antagonistically on the gene expression^36^. Although many studies indicated interaction of CKs and sugars in regulating the main physiological processes in plants^23,37^, the mechanisms by which different response pathways cross-talk are yet to be fully elucidated.

The physiological and molecular aspects of in vitro regeneration of kohlrabi (*Brassica oleracea* var. *gongylodes*) have been considerably investigated by our group^6,12,38,39^. In our previous studies, intact kohlrabi seedlings were shown as the most capable source of efficient callus-mediated de novo shoot formation on nutrient media only supplemented with CKs, excluding the need for exogenous auxins, which was accompanied by a distinctive change in endogenous levels of CKs and indole-3-acetic acid (IAA)^6^. Subsequent investigations of such regeneration system further demonstrated respective roles and expression patterns of selected organogenesis-related genes in the organogenic process throughout DNSO^12^. Additionally, our group investigated effects of different concentrations of various sugars as well as PGRs on in vitro growth and development of kohlrabi, with an emphasis on morphological alternations^39^.

While our previous work revealed that phytohormone metabolism and phytohormone-responsive organogenesis-related genes are triggered by CK signalling in DNSO of kohlrabi, the effect of sucrose on callus formation and shoot regeneration remains poorly understood in this agriculturally important crop. This study presents a follow-up of our previous phytohormonal and genetic characterisation of efficient one-step shoot regeneration from intact seedlings of kohlrabi cv. Vienna Purple. Thus, this work aims to determine the effects of both sucrose and exogenous CKs—*trans*-zeatin (*trans*Z) and thidiazuron (TDZ) on alterations in endogenous CK homeostasis and expression patterns of various organogenesis-related genes (*PIN3*, *ARR5, RGD3, CDKB2;1* and *CYCB2;4*) over the course of DNSO in kohlrabi.

## Results

### Effect of sucrose on kohlrabi growth and development during cytokinin-induced de novo shoot organogenesis

**Figure 1:**
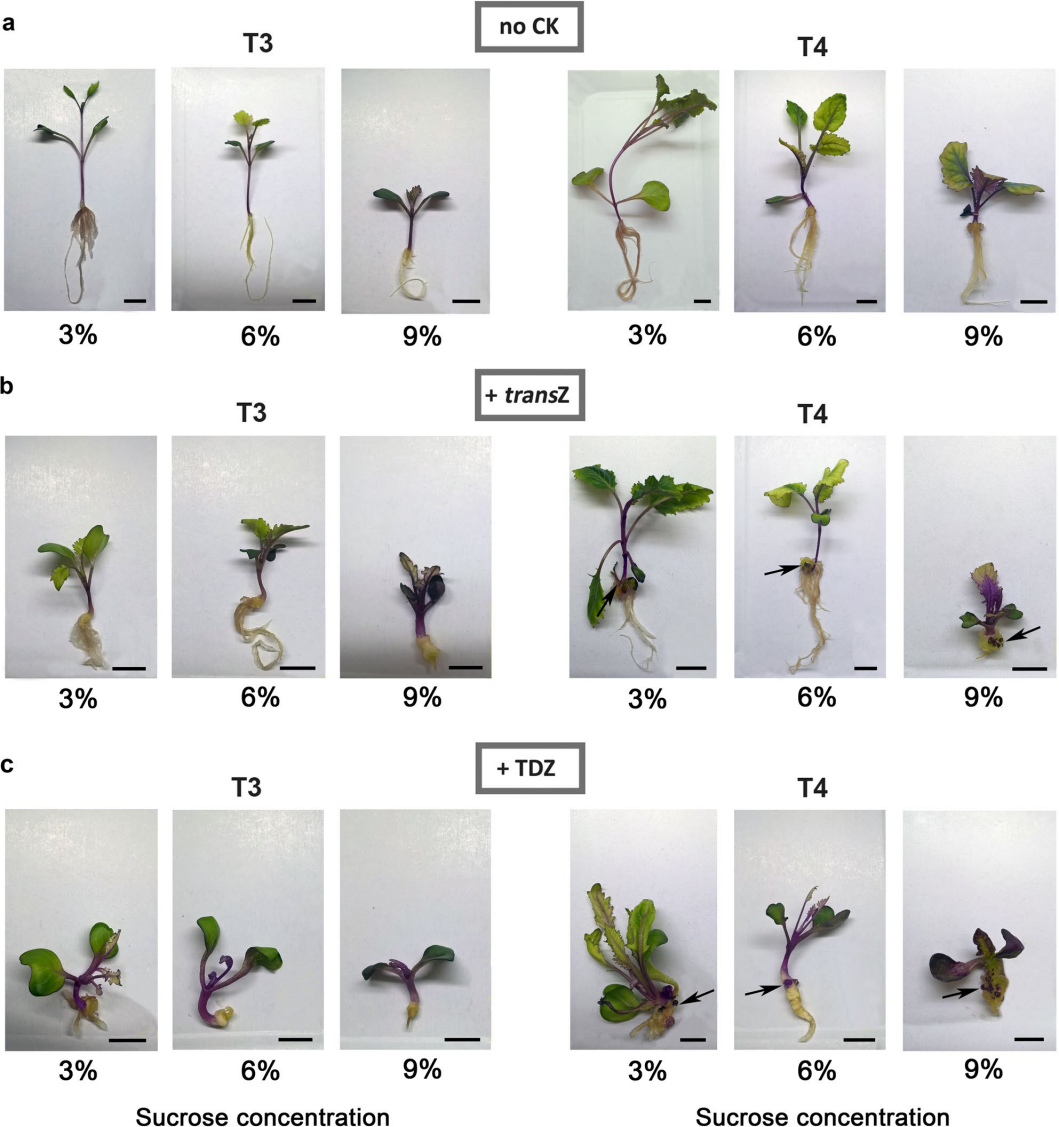
Callus formation (T3) and de novo shoots regeneration (T4) during de novo shoot organogenesis (*arrow*) of kohlrabi (cv. Vienna Purple) on media containing 3/6/9% sucrose without the presence of cytokinins (**a**), with 2 mg L^−1^ *trans*Z (**b**) or with 2 mg L^−1^ TDZ (**c**). Size bar = 1 cm. *trans*Z: *trans*-zeatin; TDZ: thidiazuron.

Optimal sucrose concentration for growth of in vitro kohlrabi cultures is 3%^6,12,38,39^. Present study demonstrates the impact of higher sucrose application (6% and 9%), decreasing the frequency of kohlrabi seed germination with the following germination rates recorded after 6 days of cultivation for investigated growth media: CK-free 78.3%, 62.5%, 40.8% (3, 6, 9% sucrose, respectively); *trans*Z 90.0%, 73.3%, 48.2% (3, 6, 9% sucrose, respectively); TDZ 81.1%, 72.5%, 53.2% (3, 6, 9% sucrose, respectively). Presence of high levels of sucrose induced alterations in kohlrabi growth as well, particularly in later stages of DNSO, marked as T3 (calli formation) and T4 (shoot regeneration) (Fig. 1). Increasing sugar concentration in CK-free growth media led to the development of shorter and thicker plantlet stem, larger root system, as well as a lower number of leaves in a dose-dependent manner (Fig. 1a), and overall caused delay in the kohlrabi development compared to standard 3% sucrose application. Addition of CKs induced DNSO, forming callus at the base of the plantlet stem followed by the appearance of de novo shoots (Fig. 1b, c). However, in combination with higher sucrose doses (6% and 9%) CKs, especially TDZ, had even more pronounced effects on plant morphology except for the root system (Fig. 1c). Moreover, TDZ induced the formation of a larger number of newly formed buds arising from the callus when sugar was applied at 9% (data not shown).

### Higher sucrose concentration impact on endogenous cytokinin profiles of kohlrabi during de novo shoot organogenesis

**Figure 2:**
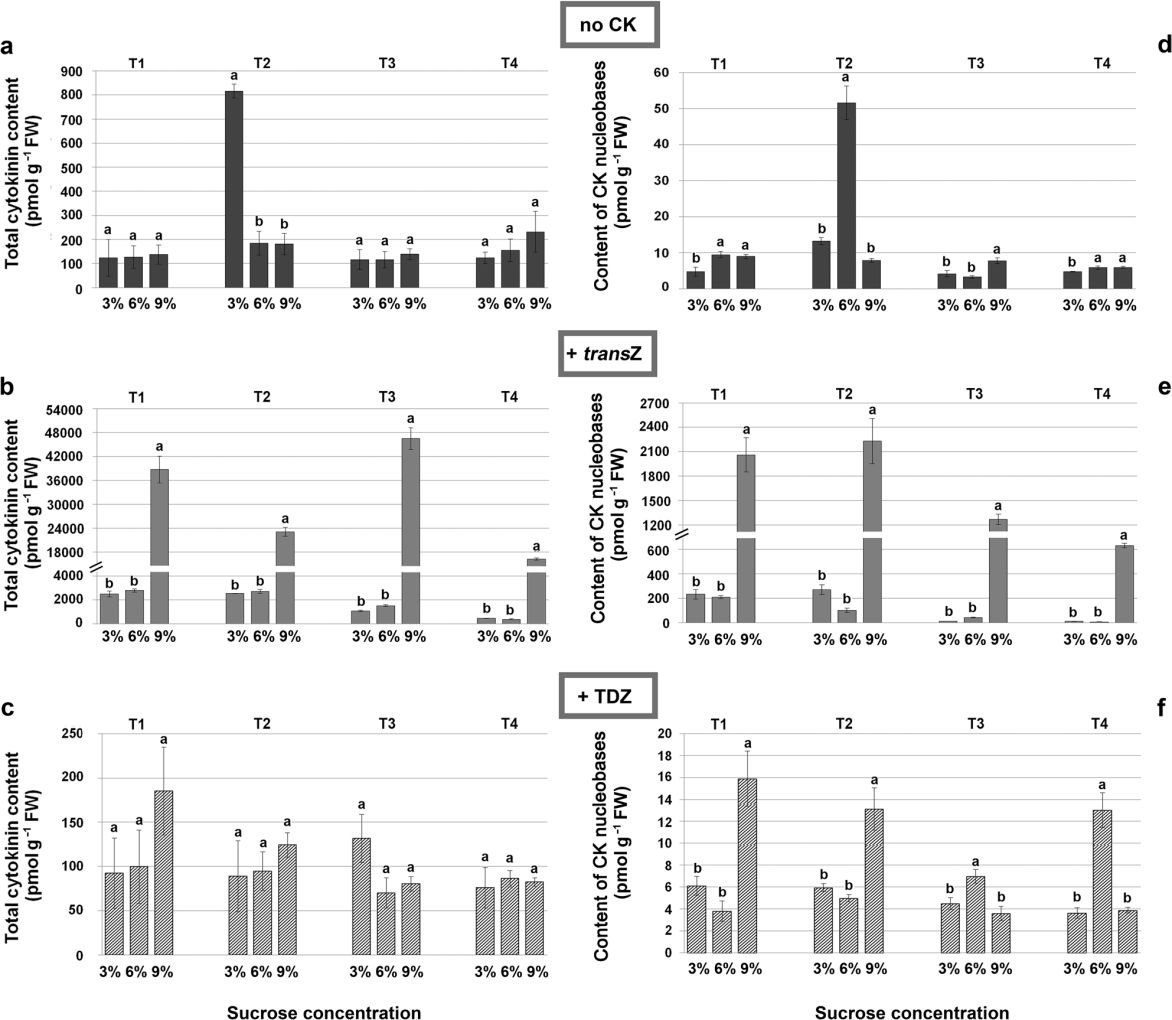
Contents (in pmol g^−1^ FW) of total cytokinins (**a**–**c**) and CK nucleobases (**d**–**f**) in four developmental stages (T1–T4) during kohlrabi (cv. Vienna Purple) growth and de novo shoot organogenesis on media containing 3/6/9% sucrose without the presence of cytokinins (**a**, **d**), with 2 mg L^−1^ *trans*Z (**b**, **e**) or with 2 mg L^−1^ TDZ (**c**, **f**). Data are shown as the mean ± SE (n = 3 independent biological replicates). Means marked with the same letter within each developmental stage of each treatment were not significantly different according to the LSD *post-hoc* test, P ≤ 0.05. *trans*Z: *trans*-zeatin; TDZ: thidiazuron.

**Figure 3:**
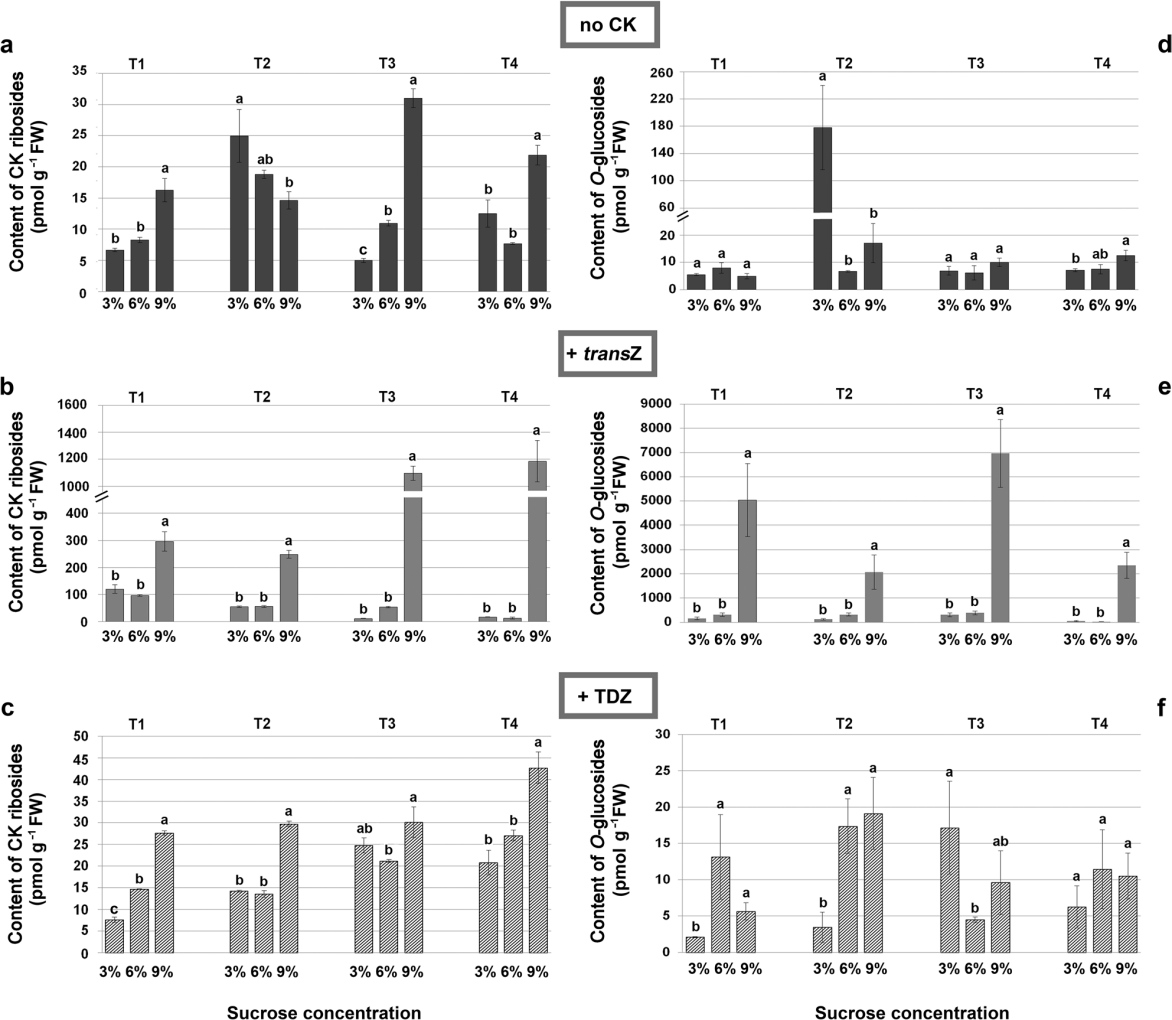
Contents (in pmol g^−1^ FW) of CK ribosides (**a**–**c**) and *O*-glucosides (**d**–**f**) in four developmental stages (T1–T4) during kohlrabi (cv. Vienna Purple) growth and de novo shoot organogenesis on media containing 3/6/9% sucrose without the presence of cytokinins (**a**, **d**), with 2 mg L^−1^ *trans*Z (**b**, **e**) or with 2 mg L^−1^ TDZ (**c**, **f**). Data are shown as the mean ± SE (n = 3 independent biological replicates). Means marked with the same letter within each developmental stage of each treatment were not significantly different according to the LSD *post-hoc* test, P ≤ 0.05. *trans*Z: *trans*-zeatin; TDZ: thidiazuron.

**Figure 4:**
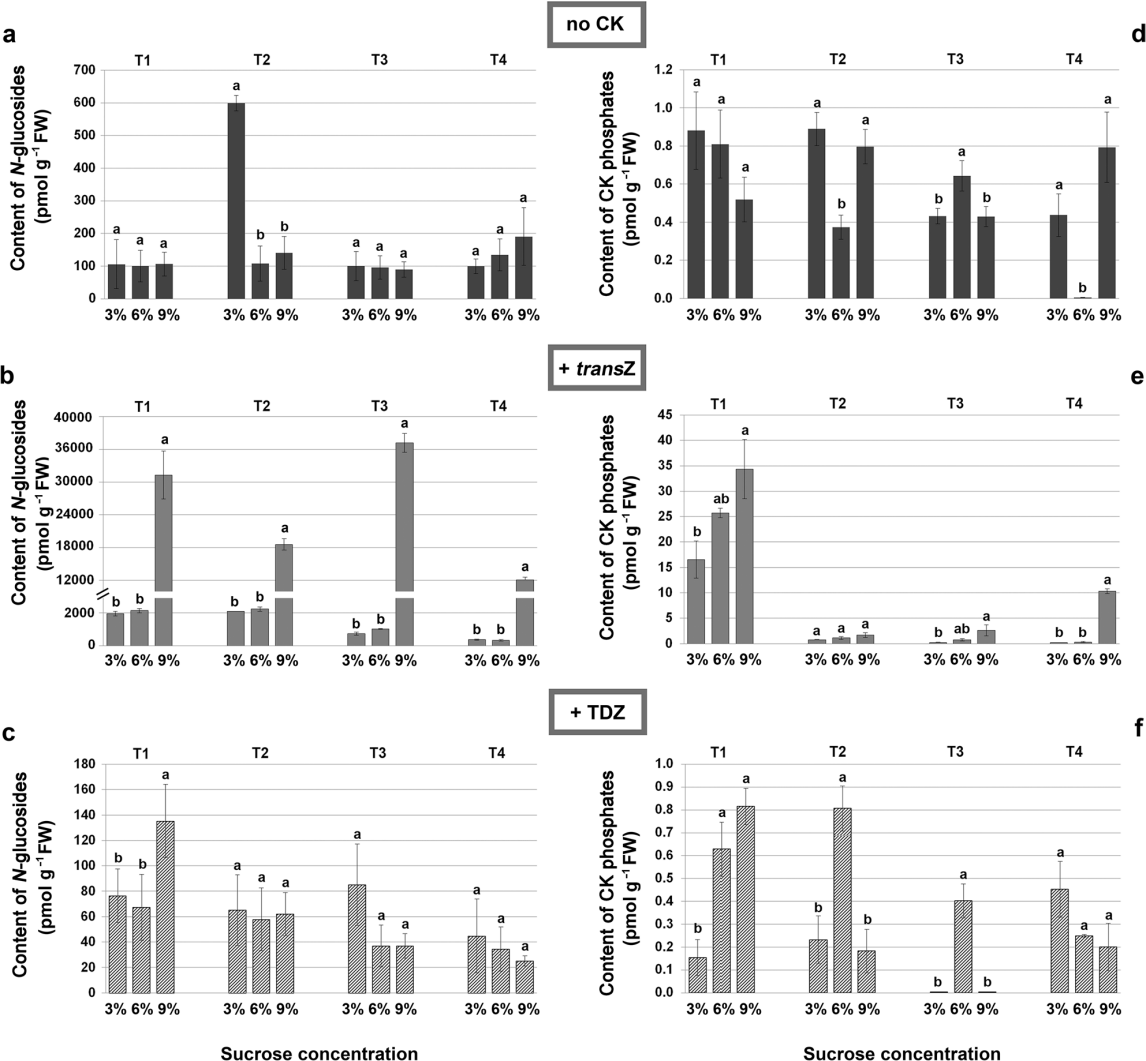
Contents (in pmol g^−1^ FW) of *N*-glucosides (**a**–**c**) and CK phosphates (**d**–**f**) in four developmental stages (T1–T4) during kohlrabi (cv. Vienna Purple) growth and de novo shoot organogenesis on media containing 3/6/9% sucrose without presence of cytokinins (**a**, **d**), with 2 mg L^−1^ *trans*Z (**b**, **e**) or with 2 mg L^−1^ TDZ (**c**, **f**). Data are shown as the mean ± SE (n = 3 independent biological replicates). Means marked with the same letter within each developmental stage of each treatment were not significantly different according to the LSD *post-hoc* test, P ≤ 0.05. *trans*Z: *trans*-zeatin; TDZ: thidiazuron.

Endogenous CK contents were investigated in kohlrabi seedlings grown on *trans*Z or TDZ enriched media with increasing concentration of sucrose and collected in four developmental stages during DNSO, including seedling development before calli formation (T1 and T2), calli formation (T3) and shoot regeneration (T4). Summary results for CKs are organised based on the CK conjugation status into five groups (Figs. 2, 3, 4): (1) CK nucleobases: *cis*-zeatin (*cis*Z), *trans*-zeatin (*trans*Z), dihydrozeatin (DHZ), *N*^*6*^-(∆^2^-isopentenyl)adenine (iP); (2) CK ribosides: *cis*-zeatin 9-riboside (*cis*ZR), *trans*-zeatin 9-riboside (*trans*ZR), dihydrozeatin 9-riboside (DHZR), *N*^*6*^-(∆^2^-isopentenyl)adenine 9-riboside (iPR); (3) *O*-glucosides: *cis*-zeatin *O*-glucoside (*cis*ZOG), *trans*-zeatin *O*-glucoside (*trans*ZOG), dihydrozeatin *O*-glucoside (DHZOG), *cis*-zeatin 9-riboside *O*-glucoside (*cis*ZROG), *trans*-zeatin 9-riboside *O*-glucoside (*trans*ZROG), dihydrozeatin 9-riboside *O*-glucoside (DHZROG); (4) *N*-glucosides: *cis*-zeatin 7-glucoside (*cis*Z7G), *trans*-zeatin 7-glucoside (*trans*Z7G), dihydrozeatin 7-glucoside (DHZ7G), *N*^*6*^-(∆^2^-isopentenyl)adenine 7-glucoside (iP7G), *cis*-zeatin 9-glucoside (*cis*Z9G), *trans*-zeatin 9-glucoside (*trans*Z9G), dihydrozeatin 9-glucoside (DHZ9G), *N*^*6*^-(∆^2^-isopentenyl)adenine 9-glucoside (iP9G); and (5) CK phosphates: *cis*-zeatin 9-riboside 5′-mono, -di, and -triphosphate (*cis*ZRMP, *cis*ZRDP, *cis*ZRTP), *trans*-zeatin 9-riboside 5′-mono, -di, and -triphosphate (*trans*ZRMP, *trans*ZRDP, *trans*ZRTP), dihydrozeatin 9-riboside 5′-mono, -di, and -triphosphate (DHZRMP, DHZRDP, DHZRTP), *N*^*6*^-(∆^2^-isopentenyl)adenosine 5′-mono, -di, and -triphosphate (iPRMP, iPRDP, iPRTP). For interpretation of the results ANOVA was used for each developmental stage point of each treatment and the system of CK abbreviations was adopted and modified according to Kamínek et al.^40^.

As shown in Fig. 2a, the total CK content was similar in all investigated developmental stages and on all sucrose concentrations applied on CK-free medium. Exception was detected for 3% sucrose in T2 stage with significantly elevated value of approximately 800 pmol g^−1^ FW compared to others ranging up to around 300 pmol g^−1^ FW. On the other hand, a remarkable increase in total CK levels was recorded in samples treated with *trans*Z, particularly when 9% sucrose was used in the medium, reaching up to 50,000 pmol g^−1^ FW in T3 (Fig. 2b). This aspect could be seen in all stages. Addition of a synthetic urea-type CK, TDZ, to the medium showed similar results as when CK was omitted, making no significant difference in total endogenous CK contents depending on the sucrose concentration used (Fig. 2c). Still, the measured values were noticeably lower compared to *trans*Z treatment.

According to ANOVA, values recorded for CK nucleobases differed with regard to the concentration of sucrose applied (Fig. 2d–f). Addition of 6 or 9% sucrose led to a significant increase in the portion of bioactive CK forms in total CK pool in all studied stages. Again, the effect of highest applied sugar concentration was particularly manifested when *trans*Z was added to the growth medium (Fig. 2e). The amendment of *trans*Z generally elicited remarkable rise in contents of CK nucleobases contents compared to CK-free medium, while application of TDZ contributed only to little or no increase (Fig. 2f).

Application of 9% of sucrose resulted in a significant rise in the level of CK ribosides, in all developmental stages except T2, during CK-free cultivation (Fig. 3a). After applying *trans*Z, substantially increased concentrations of CK ribosides were recorded in all T1–T4, reaching the highest values on medium with 9% of sucrose (Fig. 3b). Presence of TDZ in nutritive medium followed the previous trend of statistically significant increase when sucrose was applied in concentration of 9% but to a much lesser extent (Fig. 3c).

Furthermore, marked variations were found for *O*-glucosides (Fig. 3d–f) when kohlrabi plantlets were treated with *trans*Z (Fig. 3e). In all four stages (T1–T4) application of high sucrose concentration (9%) induced statistically significant increment in content of *O*-glucosides. As described earlier, TDZ showed no marked effect regardless of sucrose concentration (Fig. 3f).

The predominant CK forms in all analysed developmental stages were *N*-glucosides (Fig. 4a–c), whose distribution reflected the distribution of total CKs. Accordingly, application of *trans*Z directed to the significant increase in concentrations of *N*-glucosides, again especially at 9% of sucrose (Fig. 4b).

The situation for CK phosphates (Fig. 4d–f), the smallest fraction of total CKs representing immediate biosynthetic precursors, was different compared to other CK types. When CK-free medium was used, variations were mainly detected in combination with 6% sucrose where the CK phosphate content was reduced in T2 and T4 (Fig. 4d). Presence of *trans*Z led to the marked enhancement of these CK forms level in T1 compared to CK-free medium, mainly when 9% of sucrose was applied (Fig. 4e). On the other hand, TDZ had a diverse effect in combination with different concentrations of sugar in all studied developmental stages (Fig. 4f).

Table 1 demonstrates the distribution of endogenous CKs based on their side-chain structure into four types^41^: *cis*Z-types (*cis*Z, *cis*ZR, *cis*Z7G, *cis*Z9G, *cis*ZOG, *cis*ZROG, *cis*ZRMP); *trans*Z-types (*trans*Z, *trans*ZR, *trans*Z7G, *trans*Z9G, *trans*ZOG, *trans*ZROG, *trans*ZRMP); DHZ-types (DHZ, DHZR, DHZ7G, DHZ9G, DHZOG, DHZROG, DHZMP) and iP-types (iP, iPR, iP7G, iP9G, iPRMP). Statistical analysis showed that the levels of listed endogenous CK types in all developmental stages were generally dependent on the type of CK treatment and the applied concentration of sucrose. For all CK structural groups, the same trend was observed as previously described for the above-mentioned CK categories based on the conjugation status. The obtained CK profiles revealed a remarkable increase in endogenous CK content in samples treated with *trans*Z, mainly when 9% sucrose was used. The highest impact of exogenously added *trans*Z and high sucrose concentration was recorded for endogenous *trans*Z- and DHZ-type CKs, unsurprisingly. On the other hand, application of TDZ contributed only to little or no increase in the endogenous CK levels, regardless of the sucrose concentration, and even led to the decline of CK content.

**Table 1:** Cytokinin contents (in pmol g^−1^ FW) in four developmental stages (T1-T4) during kohlrabi (cv. Vienna Purple) growth and de novo shoot organogenesis on media containing different cytokinins and increasing concentration of sucrose. Endogenous cytokinins are divided into 4 groups based on their side chain structure: *cis*Z-, *trans*Z-, DHZ- and iP-types.

Cytokinin treatment	Sugar conc. (%)	CK content (pmol g^−1^ FW)			
		*cis*Z type	*trans*Z type	DHZ type	iP type
**T1**					
No CK	3	96.84 ± 74.28 a	9.09 ± 1.45 b	2.55 ± 0.50 a	15.34 ± 0.97 c
	6	71.45 ± 48.88 a	14.28 ± 2.78 ab	2.44 ± 0.25 a	38.40 ± 0.37b
	9	63.85 ± 36.65 a	17.64 ± 1.95 a	4.09 ± 0.94 a	51.60 ± 2.32 a
* trans*Z	3	41.80 ± 8.75 b	2247.70 ± 231.64 b	166.43 ± 21.16 b	29.35 ± 2.51 b
	6	64.06 ± 19.72 ab	2376.38 ± 131.90 b	319.70 ± 39.30 b	29.08 ± 1.67 b
	9	228.30 ± 87.05 a	35,140.84 ± 3468.62 a	3278.25 ± 197.90 a	78.60 ± 5.71 a
TDZ	3	60.93 ± 40.68 a	7.67 ± 0.73 b	4.11 ± 0.39 a	19.70 ± 1.05 b
	6	68.17 ± 43.23 a	8.27 ± 0.21 b	6.52 ± 2.84 a	16.65 ± 1.08 b
	9	94.18 ± 57.81 a	45.60 ± 4.91a	3.75 ± 0.78 a	41.77 ± 2.77 a
**T2**					
No CK	3	67.03 ± 30.97 a	638.21 ± 41.52 a	84.22 ± 16.14 a	26.79 ± 2.46 c
	6	125.25 ± 50.63 a	20.93 ± 1.06 b	1.80 ± 0.47 b	37.33 ± 0.52 b
	9	89.60 ± 44.95 a	26.89 ± 0.84 b	7.29 ± 2.35 b	57.24 ± 2.77 a
* trans*Z	3	102.78 ± 34.22 ab	2311.24 ± 22.96 b	98.53 ± 12.76 b	31.94 ± 1.53 b
	6	35.64 ± 4.99 b	2384.89 ± 128.18 b	274.48 ± 38.82 b	16.99 ± 1.42 c
	9	269.89 ± 85.43 a	21,013.27 ± 1028.41 a	1798.27 ± 218.80 a	52.87 ± 3.59 a
TDZ	3	58.91 ± 40.57 a	7.01 ± 2.11 c	2.41 ± 0.72 a	20.68 ± 4.94 a
	6	56.07 ± 24.62 a	15.21 ± 0.40 b	5.85 ± 2.04 a	17.44 ± 0.63 a
	9	73.24 ± 13.59 a	25.00 ± 2.45 a	1.96 ± 0.40 a	23.99 ± 1.97 a
**T3**					
No CK	3	69.15 ± 45.01 a	15.42 ± 1.57 b	2.36 ± 0.30 b	29.96 ± 1.47 c
	6	59.84 ± 32.13 a	16.37 ± 0.53 b	1.41 ± 0.24 c	38.82 ± 2.53 b
	9	61.02 ± 22.96 a	22.55 ± 1.25 a	3.21 ± 0.13 a	52.01 ± 2.50 a
* trans*Z	3	80.94 ± 57.41 b	772.96 ± 60.19 b	197.06 ± 29.13 b	14.54 ± 0.61 b
	6	49.36 ± 21.34 b	1023.35 ± 46.65 b	406.24 ± 58.43 b	26.73 ± 2.53 b
	9	386.34 ± 20.75 a	39,099.20 ± 2418.814 a	6952.81 ± 1174.29 a	84.04 ± 21.29 a
TDZ	3	60.99 ± 36.34 a	32.07 ± 7.22 a	1.60 ± 0.38 a	36.90 ± 2.23 b
	6	41.02 ± 16.98 a	8.40 ± 0.36 b	4.46 ± 1.59 a	16.15 ± 1.82 b
	9	43.22 ± 12.01 a	13.53 ± 4.13 b	1.75 ± 0.86 a	21.78 ± 1.13 a
**T4**					
No CK	3	46.85 ± 27.61a	39.50 ± 2.68 a	2.13 ± 0.22 a	36.01 ± 1.22 c
	6	74.31 ± 49.59 a	23.21 ± 0.25 b	1.93 ± 0.31 a	56.27 ± 4.78 b
	9	126.09 ± 85.78 a	25.93 ± 2.22 b	2.63 ± 0.29 a	77.06 ± 3.26 a
* trans*Z	3	84.33 ± 33.71 a	259.19 ± 9.91 b	72.15 ± 16.26 b	26.53 ± 1.13 b
	6	73.82 ± 41.74 a	264.72 ± 6.43 b	7.60 ± 0.17 b	21.19 ± 0.40 b
	9	328.59 ± 153.74 a	13,799.69 ± 371.035 a	23,042.35 ± 226.27 a	46.98 ± 3.37 a
TDZ	3	52.66 ± 26.16 a	6.01 ± 1.52 b	1.07 ± 0.22 b	16.18 ± 1.47 a
	6	45.12 ± 17.37 a	10.30 ± 1.41 ab	14.06 ± 5.90 a	16.75 ± 1.09 a
	9	48.40 ± 7.71 a	16.94 ± 3.12 a	1.73 ± 0.27 b	16.36 ± 0.40 a

Results are presented as mean ± SE (n = 3). Different letters indicate statistically significant difference between means within a column for each developmental stage and for each CK treatment according to Fisher’s Least Significant Difference (LSD) test, P ≤ 0.05.

(*trans*Z = *trans*-zeatin 2 mg L^−1^, TDZ = thidiazuron 2 mg L^−1^).

### Effect of sucrose on the expression of organogenesis-related genes during cytokinin-induced de novo shoot organogenesis

**Figure 5:**
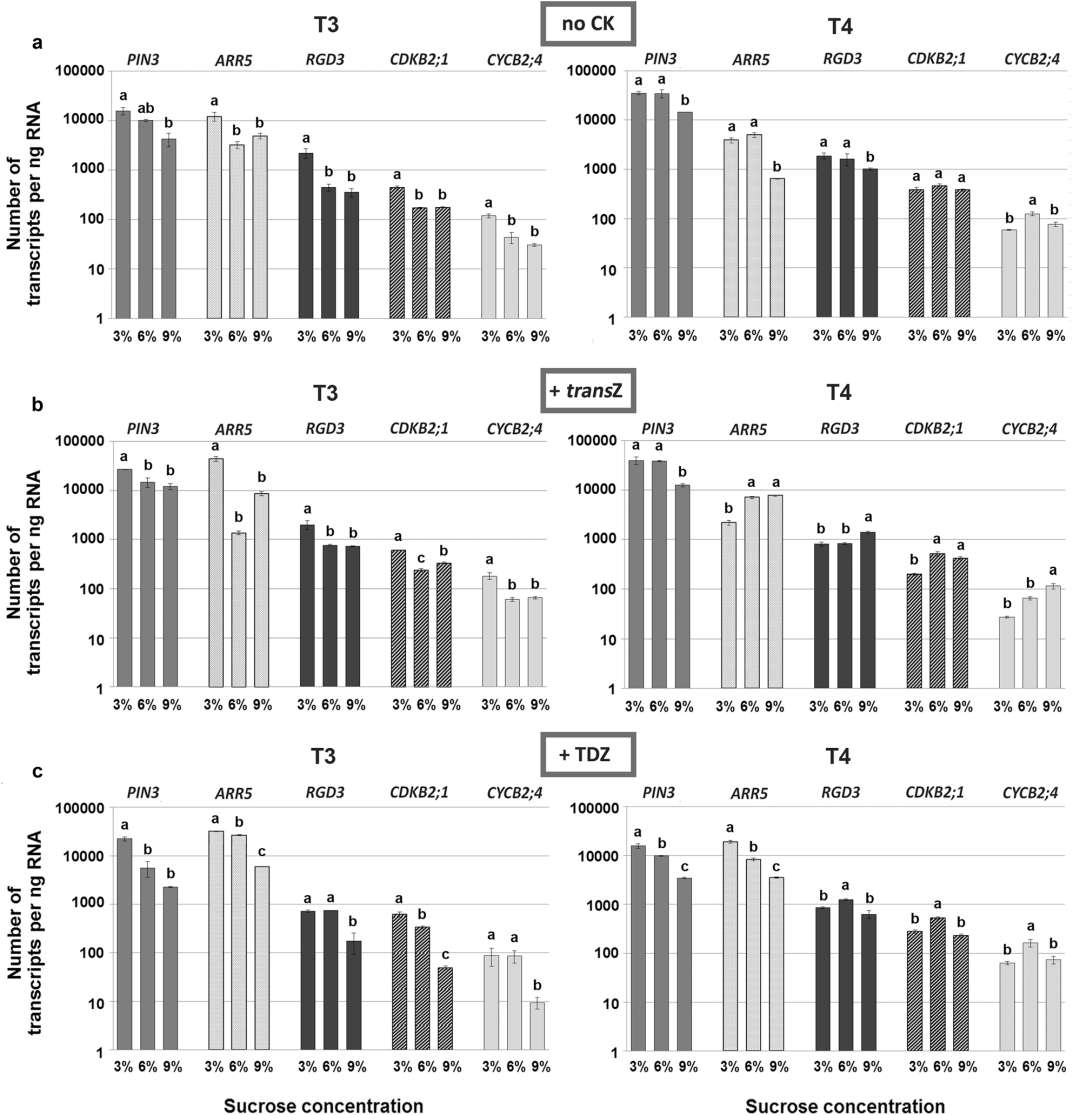
Absolute expression profiles of *PIN3*, *ARR5*, *RGD3*, *CDKB2;1* and *CYCB2;4* genes in later developmental stages (T3 and T4) during kohlrabi (cv. Vienna Purple) growth and de novo shoot organogenesis on media containing 3/6/9% sucrose without cytokinins (**a**), with 2 mg L^−1^ *trans*Z (**b**) or with 2 mg L^−1^ TDZ (**c**). Data are shown as the mean ± SE (n = 3 independent biological replicates). Means marked with the same letter for each distinct gene were not significantly different according to the LSD *post-hoc* test, P ≤ 0.05. *trans*Z: *trans*-zeatin; TDZ: thidiazuron.

The activity of selected genes involved in auxin transport (*PIN3*), CK response (*ARR5*), de novo shoot apical meristem formation (*RGD3*) and cell division (*CDKB2;1* and *CYCB2;4*) was determined in kohlrabi plantlets using quantitative PCR analysis showing variation in expression levels during callus formation (T3) and de novo shoot regeneration (T4) (Fig. 5a–c).

Absolute quantification determined for kohlrabi plants grown on 3/6/9% sucrose without CKs was performed to study the effect of sugar alone on the activity of chosen genes. The analysis revealed that a higher concentration of sucrose had a statistically significant impact on the expression of all investigated genes, diminishing their activity, particularly during callus formation. The same trend was observed in *trans*Z-treated plants in T3. On the other hand, the addition of *trans*Z induced contrary effect in combination with high sugar concentration in T4 stage leading to increased activity of investigated genes, except for *PIN3* (Fig. 5b). Application of TDZ induced a statistically significant decrease in the gene expression when higher sugar concentration was used, particularly 9% in T3 (Fig. 5c). The *RGD3*, *CDKB2;1*, and *CYCB2;4* genes showed slightly different expression patterns in T4, with 6% sucrose being the treatment with the highest values for absolute expression.

### Correlations between endogenous cytokinins and activity of the analysed genes during later stages of de novo shoot formation

**Figure 6:**
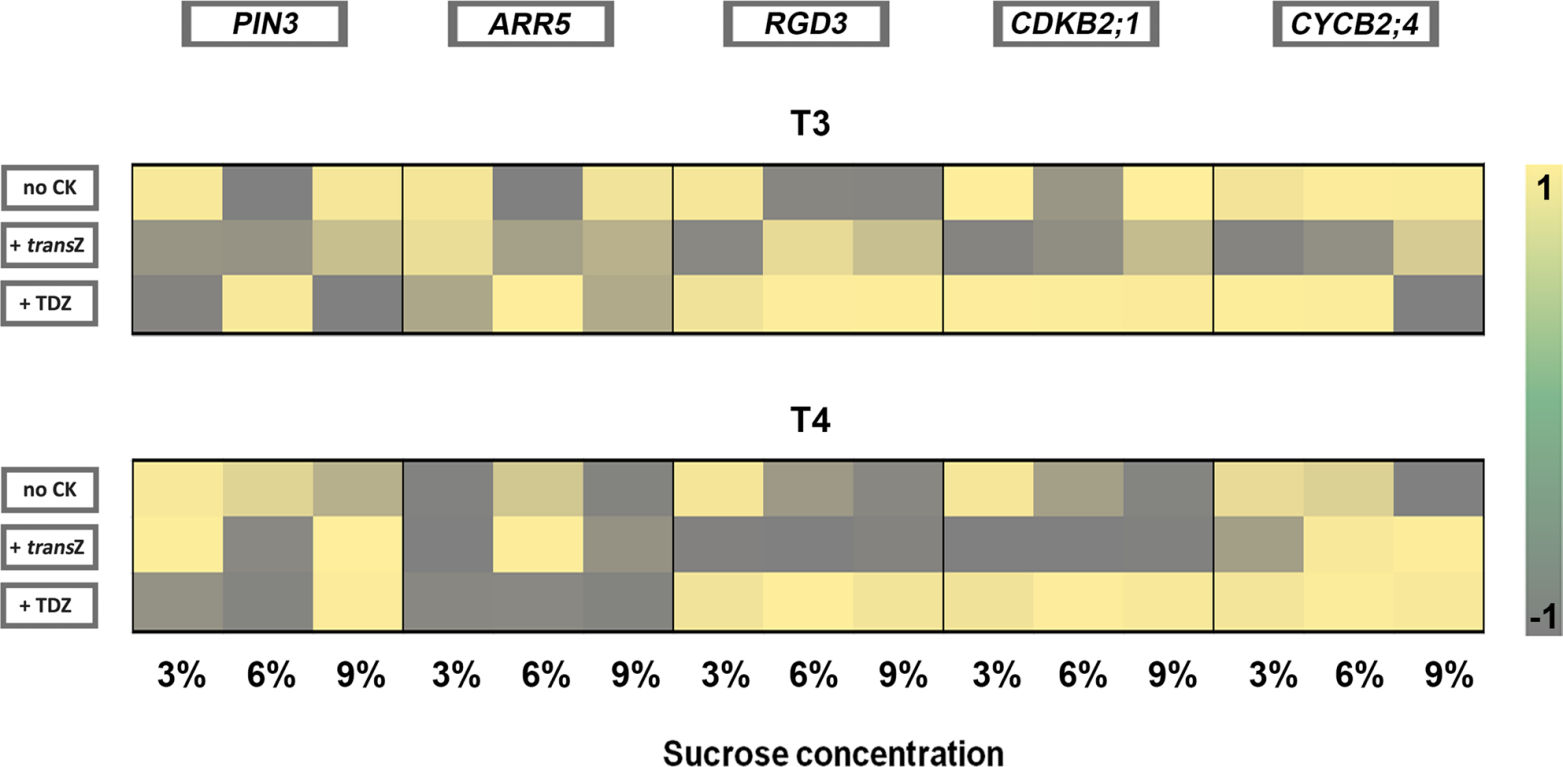
Heat map representing Pearson’s correlation coefficient (r) calculated between content of CK nucleobases and absolute gene expression of differently treated kohlrabi (cv. Vienna Purple) seedlings for T3 and T4 stages of de novo shoot organogenesis.

In order to investigate if altered endogenous CK homeostasis affected the activity of given organogenesis-related genes, correlations between content of CK nucleobases, representing bioactive CKs, and absolute expression of analysed genes were evaluated in response to each particular CK/sucrose treatment, at the T3 and T4 stages of organogenesis. As demonstrated in Fig. 6, at the T3 stage of organogenesis, there is positive correlation between CK nucleobases and expression of most genes at 3% and 9% sucrose treatments. When it comes to *trans*Z treatments, correlation between bioactive CKs and the expression of most genes is negative at 3% sucrose, but this negative correlation becomes less pronounced with increasing sucrose concentration in the media. At all TDZ treatments, there is strong positive correlation between bioactive CKs and the expression of *RGD3* and the cell cycle genes *CDKB2;1* and *CYCB2;4*, with the exception of *CYCB2;4* at 9% sucrose, where the correlation shifts from strongly positive to strongly negative.

At the T4 stage of organogenesis, the situation is somewhat different. On media without added CKs, the correlation between the plant content of bioactive CKs and the expression of the investigated genes is dependent on sucrose in the media, and, with the exception of *ARR5*, shifts from strongly positive at 3% sucrose, to strongly negative at 9%. It is noteworthy that unlike with the other genes, the expression of *ARR5* is remarkably negatively correlated with the plant bioactive CK content at almost all the CK/sucrose treatments during the T4 phase. Besides, at the *trans*Z treatment, the expression of *RGD3* and *CDKB2;1* is strongly negatively correlated with the plant bioactive CKs regardless of sucrose concentration. On the contrary, at the TDZ treatment, the expression of *RGD3* and both examined cell cycle genes is strongly positively correlated with plant CK content regardless of sucrose concentration, whereas the expression of *PIN3* shows negative correlation with plant bioactive CKs at moderate sucrose concentrations, but this correlation becomes strongly positive at 9% sucrose (Fig. 6.).

## Discussion

Sugar levels influence plant development, including various processes from embryogenesis to senescence^42^. In emerging plant embryos, glucose and sucrose are involved in regulation of cell division, cell expansion as well as accumulation of reserve carbohydrate^43^. Furthermore, sucrose is implicated in cell division and starch synthesis in mature plant cells^44^. This impact is accomplished due to the fact that sugars act as signalling molecules and can regulate the expression of various genes^24^.

Plant shoots grown in vitro are characterised by a reduced ability of photosynthesis, so as compensation carbon source must be included in the nutritive media^45^. The most exploited sugar for this purpose is sucrose, which besides being an energy source also plays a significant role as osmotic regulator.

The optimal concentration of sucrose in shoot cultures is dependent not only on the plant species but also on different genotypes and cultivars^46^. Our previous work demonstrated that kohlrabi in vitro cultures grow well on an optimal concentration of 3% sucrose^6,12,38,39^, which enables an efficient regeneration process. In the present study we investigated the application of 6% and 9% sucrose on kohlrabi germination, seedling growth and regeneration. The results were obtained showing a proportional decrease in germination rate as a response to the increased amount of sugar in the medium after six days, regardless of exogenous CK addition. Other studies previously demonstrated the sugar-induced delay of seed germination, indicating involvement of abscisic acid (ABA); namely it was suggested that elimination of endogenous ABA is slower in seeds germinating under the influence of sugar, hence the delay^47^.

Similarly, further growth and development of kohlrabi seedlings were affected by the presence of a higher amount of sucrose—the development of shorter and thicker plantlet stem, larger root system, as well as a lower number of leaves were recorded in a dose-dependent manner. Described growth changes are in accordance with our former work where growth and development of treated kohlrabi seedlings were significantly affected due to sugar treatment in a distinctive manner, with a variety of morphological traits being altered in comparison to matching controls^39^.

Seedlings germinated on media with higher concentrations of sugars, such as glucose or sucrose, usually have shorter hypocotyls whose length is inversely proportional to applied sugar concentration, less developed root system and leaves^48,49^. On the other hand, in *Brassica napus* seedlings application of exogenous sucrose led to inhibition of hypocotyl growth while it stimulated the root growth^31^, as in our study with kohlrabi plantlets grown on CK-free media. Improved root growth under a high sucrose level (6%, w/v) was also reported in *Arabidopsis* seedlings overexpressing melon tonoplast sugar transporter CmTST1^50^. Authors proposed that CmTST1 plays a key role in importing sugar into the vacuoles of roots in response to metabolic demands to maintain cytosolic sugar homeostasis.

Different concentrations of constituents of nutritive media affect morphogenesis and growth of the cultured plant cells, tissues and organs partially due to their nutritional value but can also alter its osmotic potential^51^. Stress-shocked cells showed cell growth retardation and the induction of plasmolysis^52^. Biahoua and Bonneau^53^ reported in their study of the spindle tree somatic embryogenesis that sucrose played an osmotic role stimulating plant regeneration rather than acting exclusively as a carbohydrate source. It was estimated that larger part of the applied sucrose that was needed for inducing an optimal rate of adventitious shoot formation in hypocotyl explants of *Digitalis obscura* were required as energy source, while the rest osmotically regulated morphogenesis^54^.

Observed developmental alterations and growth retardation were enhanced by the presence of CKs, so they might not represent a primary consequence of osmotic stress but could be instead associated to sucrose crosstalk with other response pathways such as e.g. phytohormone response pathways. Sugar sensing processes and their effects on plant development arrest are already shown to be separated from their nutritive role^55^.

We can assume that supra-optimal sucrose combined with exogenous CKs largely contributed to the stunted and deformed kohlrabi phenotypes, by promoting excessive stimulation. TDZ manifested even stronger effect than *trans*Z, and also induced the formation of a larger number of newly formed buds when sugar was applied at 9%. It is widely considered that synthetic substances with cytokinin-like biological effects (such as TDZ) have additional ways of influencing plant morphogenesis, that are not typically attributed to isoprenoid cytokinins (such as *trans*Z)^56,57^. In some cases higher concentrations or prolonged treatment of TDZ could obstruct further growth and development of the regenerants, which might represent the consequences of TDZ effect on metabolism of endogenous auxin and cytokinin^58,59^. On the other hand, these morphological changes could be the result of alterations in kinetics of plant enzymes, as many of the TDZ-stimulated enzymes were shown to be associated with cell walls, membranes and membrane fluidity^60^. Thus, TDZ might influence morphological alterations that are qualitatively distinct from those induced by zeatin, and not necessarily related to the dynamics of DNSO as described in the standard two-step shoot regeneration protocol for *Arabidopsis*.

Levels of endogenous phytohormones are influenced not only by plant species and genotypes but also by the type of explant and the developmental stage^6,61^. As previously shown, the presence of PGRs in nutritive media affects the content of endogenous plant hormones through alteration of their homeostasis^5,6^.

Although the total CK content in in vitro grown kohlrabi plantlets was not affected by higher sugar application alone, a substantial rise in the CK level was documented after *trans*Z application together with 9% sucrose regardless the developmental/DNSO stage. Our earlier report on kohlrabi DNSO demonstrated that after six weeks of seedling cultivation on different CKs, exogenously applied *trans*Z into the medium with 3% sucrose contributed not only to the notable increase of endogenous levels of zeatin-type CKs but also to the enhancement of the total CK pool^6^. The stadium of analysed kohlrabi samples in that work approximately corresponded to the T4 stage in the present study (emergence of de novo shoots), with comparable total CK level on 3% sucrose (441 pmol g^−1^ FW). We assume that the CKs from regeneration media affect endogenous levels of particular CK derivatives upon uptake from the media, ultimately contributing to the rise in their content, as previously reported^4,41,62^.

In this regard, the mechanism by which high sucrose concentration enhances the plant CK levels specifically in combination with *trans*Z is particularly intriguing. This increase is presumably a consequence of the uptake from nutrition media, upon which CKs may either stay in the form of free bases, or undergo conjugation into other CK types, as reflected in the increase of all CK types (organised based on the CK conjugation status) on the *trans*Z/9% sucrose treatment. The accumulation of these CK classes, particularly at 9% of sucrose, could be not only an implication of the increased uptake, but also of the disturbed hormonal homeostasis and, consequently, metabolic glucosylation^4,62,63^. Overaccumulation of sugars due to higher carbohydrate supply might have altered the metabolic processes in the plant cells, which subsequently altered the CK levels and ultimately growth and development of plantlets. It was shown that plants display improved acclimation to osmotic stresses when endogenous CK content is high. Elevated CK levels under drought stress neutralize drought-induced signalling, which enables plants to preserve normal levels of leaf water content, photosynthetic rate and stomatal conductance under stress conditions^64^. On the other hand, different studies showed that sugars control the biosynthesis, transport, or signalling of particular hormones, including auxin and cytokinins^23,65,66^. It was suggested that sugars promoted cytokinin accumulation through upregulation of some of the *IPT* genes^23,37^.

The elevation of CK levels in plantlets grown on media with 9% sucrose was identified in all four investigated developmental stages during DNSO, with the maximum increment observed for T3 stage characteristic by callus formation at the base of the kohlrabi plantlets. The detected values were ca. 40-fold to over 100-fold higher for total and bioactive CKs, respectively, compared to control (3% sucrose). Earlier literature data suggested that plant phytohormone signalling depended exclusively on the levels of endogenous hormones occurring in plant tissues, which may not relate to the PGRs present in the regeneration media^67^. However, it was shown that both exogenous and endogenous plant hormones could initiate the callus formation and subsequent cell differentiation in plant tissue culture, interacting with each other to induce shoot organogenesis^68^.

Similar situation was found when endogenous CKs were classified based on their *N*^6^ side-chain structure (Table 1). Considering this categorisation, higher concentration of sucrose synergistically acted with *trans*Z mostly affecting *trans*Z-type CKs, upraising their levels up to nearly 40,000 pmol g^−1^ FW in T3.

The lowest contribution of exogenously applied *trans*Z to the increase of endogenous levels was noticed for iP-type CKs considered primary precursors for biosynthesis of zeatin-types^69^. The decrease of iP-type CKs in *trans*Z-treated plants suggests that enhanced uptake of *trans*Z might have triggered homeostatic mechanisms that lead to the decrease in the levels of its biosynthetic precursor and related conjugates, consistently with our previous findings^6^. However, it should be mentioned that application of 9% of sucrose, without any PGRs also induced elevation of iP-types compared to control on 3% in all stages, suggesting the interference of sugar with CK homeostasis.

Accordingly with our previous work^6^, the presence of TDZ in the shoot regeneration media did not significantly affect the endogenous levels of any of CK groups, classified based on their *N*^6^ side-chain structure, as compared to the control regardless of sucrose concentration. Earlier literature data showed that treating plant tissues with synthetic CK homologues initiated at least a transitory elevation in levels of natural zeatin, and zeatin riboside^70^. It has been known for a long time that TDZ has an inhibitory effect on CK catabolism through the inhibition of CK oxidase/dehydrogenase (CKX)^71^. Our results show that although it did not generally contribute to an increase in plant CK levels, TDZ affected the signalling of organogenesis-related genes.

On the other hand, the level of applied sugar seems to be important cofactor with TDZ affecting some CK groups, such as CK nucleobases and ribosides, as well as phosphates. Thidiazuron is a synthetic derivate of phenylurea with proven effects similar to CKs. Although it structurally differs from both isoprenoid and aromatic CKs, it possesses the ability to efficiently bind CK receptors^72^. However, along with indisputable CK-like activities, TDZ can also manifest some other biological effects on plant tissues, such as auxin-like ones^56^. Furthermore, the capacity of TDZ to provoke a defensive response in plant tissues can also initiate down- or up regulation of other phytohormones and secondary metabolites, and at the same time moderate the influx/efflux of specific cations, such as calcium, through biological membranes^56,73,74^. TDZ, as a competitive inhibitor of CKX^71^ can affect pathways of purines and cytokinin metabolites by preventing their breakdown^56^. Despite the range of effects associated to TDZ and intensive research, its precise mode of action in induction of in vitro plant morphogenesis has not been fully clarified to date^56,57^.

Kiba et al.^75^ suggested that de novo biosynthesis of CKs is triggered by photosynthetically generated sugars in *Arabidopsis* grown in elevated CO_2_ conditions. Their research has indicated the existence of a signalling pathway in which the photosynthesis-derived sugars regulate CK levels to control plant growth and development. Earlier transcriptome analysis revealed that sugar treatments upregulate *AtIPT3* and *CYP735A2* genes^36,76^ suggesting that sugar-induced expression of genes is implicated in de novo CK biosynthesis.

The expression of *IPT*, and consequently CK levels are influenced by the accessibility of macronutrients like nitrogen, phosphate and sulphate^77–79^. Considering that macronutrients and carbon metabolism are closely linked, one can hypothesise that sugars regulate expression of genes involved in the de novo biosynthesis of CKs indirectly via signalling pathways of macronutrients^75^.

Some reports indicate that sugars and CKs can function both agonistically and antagonistically on gene expression^23,36^. The antagonistic effect of CKs was demonstrated through direct interaction between sugar and CKs using *hxk1/gin2* mutant^32^. However, other studies from the same research group demonstrated synergistic effects as reported for *Arabidopsis* seedlings, where CKs and glucose positively regulated root elongation through an HXK1-dependent pathway^80^. This response implicated cytokinin receptor AHK4 (ARABIDOPSIS HISTIDINE KINASE 4) as well as three type B response regulators (ARR1, ARR10, and ARR11) that are positive regulators of CK signalling pathway, with glucose enhancing CK-dependent root elongation upstream of the auxin effect on root growth^23,80^.

It was found that glucose influences the expression of a majority of genes involved in CK metabolism, perception and signalling. Upregulation of *AtIPT3* by glucose indicates the possibility of glucose upregulating CK biosynthesis. Additionally, glucose upregulates specific components of CK signalling, namely A-type response regulators, which function as negative regulators of CK signalling pathway^81^, and CK response factors (CRFs)^36^. This kind of glucose-modulated expression of certain components of CK signalling suggests that sugar and CK signalling can interact with each other, with sugars presumably affecting CK homeostasis from biosynthesis to signalling.

Currently it remains unclear whether transporter activity, membrane damage, osmotic stress or specific signals underlie observed remarkable increase in CK content when exposed to high sucrose/*trans*Z combination. The phenotypic flexibility of plant growth and development is a result of signalling network that merges signals, both from inside and outside, and comprises nutrients, phytohormones, as well as physical factors. Besides, identical signals can stimulate diverse responses at different concentrations, making the phenomenon of sugar sensing even more complicated. To provide clear evidences supporting sucrose effect on the CK homeostasis detailed expression analyses of genes regulating CK levels in planta need to be performed.

Investigation of sugar effects and endogenous CK levels during DNSO in kohlrabi seedlings in vitro was complemented with determinations of absolute expression profiles of organogenesis-related genes involved in auxin transport, CK response, de novo shoot apical meristem formation and cell division. The selected genes were previously shown to be essential elements of CK signalling cascade in the course of de novo formation of shoots in established efficient kohlrabi regenerating system^12^. Application of sucrose in high concentration without the presence of exogenous CKs resulted in a significantly decreased expression of all investigated genes during callus and de novo shoot development. This decrease could be associated with the observed retardation in kohlrabi development compared to standard 3% sucrose treatment.

It has been shown in our previous work^12^ that these genes are upregulated during callus formation (T3) on media with 3% sucrose; their upregulation is most likely closely linked to the molecular processes that accompany callus formation^9,12^. Here we demonstrate that increased concentration of sucrose in regeneration media causes downregulation of these genes compared to 3% sucrose, likely accounting for the delay in morphogenic events that accompanied DNSO in culture.

The same effect of sucrose is present for the genes *PIN3* and *ARR5* during the T4 regeneration phase (shoot formation), whereas it is not evident, or in some cases even reversed, for *RGD3* and the cell cycle genes *CDKB2;1* and *CYCB2;4*. These genes are predominantly downregulated during the shoot formation phase on media with 3% sucrose^12^, consistently with a different expression pattern and different response to sucrose compared to their expression profiles during T3.

Pearson’s correlation analysis revealed interesting features of the correlation between plant bioactive CK levels and the expression of organogenesis-related genes in various exogenous CK/sucrose treatments. An interesting phenomenon is negative correlation between bioactive CK levels and expression levels of most investigated genes, in most of the *trans*Z treatments, in both T3 and T4 organogenesis stages. As all these genes were shown to be CK-responsive^12^, a highly positive correlation between plant CK levels and their expression would be expected; yet, we demonstrate that the opposite is mostly the case. A possible explanation lies in very high plant CK levels that accompany the *trans*Z treatment; as a response to excessive presence of the hormone, signalling may be silenced by homeostatic mechanisms, resulting in negative instead of positive correlation between the plant CK levels and gene expression. Similarly, a strong negative correlation is present during the T3 phase of organogenesis in hormone-free media with 6% sucrose, where endogenous CK levels were particularly high during the previous T2 phase.

The correlation analysis also showed that during the T4 phase, the correlation between plant bioactive CK levels and the expression of most organogenesis-related genes depends on the concentration of sucrose in the media, being, except for *ARR5*, strongly positive at 3% sucrose and shifting towards strongly negative at 9%. This is another indication that sucrose from the nutrient media interacts—or at least interferes—with the CK-mediated regulation of these genes. The only exception is *ARR5*, whose expression levels are negatively correlated with plant CK levels during the T4 phase on almost all CK/sucrose media combinations. This is unsurprising, as *ARR5* is known to undergo a strong inhibition by CK-induced *WUSCHEL* around the time of shoot development in *Arabidopsis*^82,83^ and the downregulation of *ARR5* upon shoot development was also shown in kohlrabi in our previous work^12^.

Finally, correlation analysis also revealed interesting relations between plant CK content and organogenesis-related gene expression in TDZ treatments during the T4 stage. While the expression of *ARR5* was strongly negatively correlated with plant CK content regardless of sucrose concentration in the media, the opposite was true for *RGD3* and the two cell cycle genes *CDKB2;1* and *CYCB2;4*. Interestingly, the expression of the auxin transporter gene *PIN3* was negatively correlated with plant CK content at moderate sucrose concentrations (3% and 6%), whereas this correlation was positive on media with high sucrose, suggesting that sucrose might interfere with either TDZ or its impact on endogenous CKs affecting the expression of this auxin transporter gene.

In conclusion, we used our previously established efficient one-step DNSO system as a useful tool to investigate the effect of sugar on plant growth and organ development, and ultimately intricacy of the sugar and phytohormone signalling networks. Application of moderate sucrose concentration did not alter the CK homeostasis significantly, with results similar to control for both types of CK treatment. Contrary to that, a higher concentration of sucrose in nutritive media containing *trans*Z contributed to a substantial increase in endogenous CK levels throughout kohlrabi development and DNSO. Absolute quantification of the selected genes revealed that higher concentration of sucrose significantly declined their expression, particularly during callus formation. This trend was demonstrated for TDZ as well as *trans*Z treatment in T3. On the other hand, higher sugar concentration induced contrary effect in combination with *trans*Z in T4 stage, leading to the increase of gene expression. Correlation analysis suggests that sucrose might interfere with how plant CK levels affect the expression of organogenesis-related genes during callus and shoot formation in kohlrabi. Our results indicate that phytohormone metabolism may be affected by sucrose in kohlrabi shoot organogenesis. It should be, however, yet elucidated whether sugars act as signalling molecules, energy sources or building units to impact the expression of the particular genes. Undoubtedly, the regulatory network is more complex and must comprise a role for sugars in the regulation of hormone metabolism as well as hormone signalling.

## Methods

### Plant material and growth conditions

Establishment of kohlrabi (*Brassica oleracea* var. *gongylodes* cv. Vienna Purple) in vitro culture was performed as previously described in Ćosić et al.^6,12^. Accordingly, seeds were primarily surface sterilized by a 5 min submersion in 70% ethanol. This step was followed by 30 min submersion in 30% commercial bleach (4–6% NaOCl) containing a drop of detergent (Fairy; Procter and Gamble, London, UK) and rigorously rinsing in autoclaved distilled water. Sterilized seeds were aseptically transferred to particular growth media.

A basal, PGRs-free growth medium comprised of Murashige and Skoog^84^ mineral salts, Linsmaier and Skoog^85^ vitamins, 100 mg L^−1^ inositol and 0.6% agar. For the investigation of sucrose concentration effects, media containing 3%, 6% or 9% sucrose were used. At the same time, different regeneration media have been additionally complemented with *trans*-zeatin or thidiazuron at 2 mg L^−1^. The respective CK concentrations have been previously established through an optimization of the regeneration protocol for various explant types of kohlrabi cultivars Vienna Purple and Vienna White, as described earlier^6^. All media used in this study were adjusted to pH 5.8 with 1 N NaOH before autoclaving at 114 °C and 80 kPa for 25 min. Cultures were maintained in a growth chamber at 25 ± 2 °C under cool white fluorescent light (16 h light photoperiod; 47 μmol m^−2^ s^−1^ irradiance).

The experimental setup for evaluating the impact of sucrose on CK homeostasis and organogenesis-related genes in four different stages (T1-T4) of CK-induced DNSO in kohlrabi seedlings implied eight 375 mL jars for each specific combination of sucrose and CK. Each jar contained six surface sterilized seeds. Control seeds were grown on basal growth medium with 3% of sucrose.

Plant material was collected in four developmental stages (T1-T4), and included seedlings bearing two cotyledons (T1), plantlets with emerged two leaves (T2), plantlets forming callus at the base of the stem (T3) and de novo shoots appearing from the calli (T4). Plantlets grown on CK-free medium were collected at the same time as corresponding CK treated plantlets, having in mind that DNSO occurred only on media supplemented with *trans*Z or TDZ. For each stage point, three representative plantlets exposed to the same treatment were collected and pooled into a single biological sample. All samples were immediately frozen in liquid nitrogen and stored at -70 °C until analysis. The entire experimental setup was conducted in three independent biological replicates.

### Determination of endogenous cytokinin levels

Phytohormone analysis was carried out as described in Dobrev and Vankova^86^ and Djilianov et al.^87^. Approximately 100 mg of plant material of each biological sample was homogenised in liquid nitrogen and then lyophilised.

Cold extraction buffer consisting of methanol/formic acid/water (15/1/4; v/v/v) was added to the plant homogenates, along with a mixture of stable isotope-labelled internal standards (10 pmol). The listed internal standards were used: [^2^H_5_]*trans*-zeatin (*trans*Z); [^2^H_5_]*trans*Z-9-riboside (*t*ZR); [^2^H_5_]*trans*Z-7-glucoside (*t*Z7G); [^2^H_5_]*trans*Z-9-glucoside (*trans*Z9G); [^2^H_5_]*trans*Z-*O*–glucoside (*trans*ZOG); [^2^H_5_]*trans*ZR-*O*–glucoside (*t*ZROG); [^2^H_5_]*trans*ZR-5´-monophosphate (*t*ZRMP); [^2^H_3_]dihydrozeatin (DHZ); [^2^H_3_]DHZ-9-riboside (DHZR); [^2^H_3_]DHZ-9-glucoside (DHZ9G); [^2^H_6_]*N*^*6*^-(∆^2^-isopentenyl)adenine (iP); [^2^H_6_]*N*^*6*^-(∆^2^-isopentenyl)adenosine (iPR); [^2^H_6_]iP-7-glucoside (iP7G); [^2^H_6_]iP-9-glucoside (iP9G); [^2^H_6_]iPR-5´-monophosphate (iPRMP) (all CK standards were from OlChemIm, Olomouc, Czech Republic). The concentrations of *cis*-zeatin (*cis*Z) derivatives were determined based on the retention times and mass spectra of unlabelled standards and the response ratio of their *trans*Z counterparts.

Detection and quantification of CKs were performed using HPLC (Ultimate 3000, Dionex, Sunnyvale, CA, USA) that was coupled to a hybrid triple quadrupole/linear ion trap mass spectrometer (3200 Q TRAP, Applied Biosystems, Foster City, CA, USA) set in selected reaction-monitoring mode. The mass spectrometer was set at electrospray ionisation mode with following ion source parameters: ion source voltage + 4000 V (positive mode); nebuliser gas 50 psi; heater gas 60 psi; curtain gas 20 psi; heater gas temperature 500 °C. The endogenous CKs were quantified using the isotope dilution method with multilevel calibration curves. All data were processed with Analyst 1.5 software (Applied Biosystems). The CK concentrations were calculated as the amount per 1 g of fresh weight plant material.

For evaluation of endogenous CK levels, three biological replicates were used for each of kohlrabi seedlings treated with *trans*Z/TDZ + 3/6/9% sucrose as well as control. Analyses were repeated twice with comparable results.

### RT-qPCR analysis

Extraction of total RNA from collected kohlrabi samples was performed according to the protocol of Gasic et al.^88^ including DNase I (Thermo Scientific, Waltham, MA, USA) treatment, as previously described in Ćosić et al.^12^. Briefly, around 150 mg of each sample was ground in liquid nitrogen with the addition of CTAB extraction buffer. After two series of chloroform:isoamyl alcohol (24:1, v/v) extraction and overnight precipitation, final RNA precipitates were resuspended in RNase-free water. Total RNA quantity and quality were determined using the standard procedures of UV absorption spectrophotometry and gel electrophoresis.

RevertAid reverse transcriptase with random hexamer primers (Thermo Scientific) was used for performing reverse transcription (RT), with reactions arranged according to the manufacturer’s protocol, each containing 0.5 μg RNA. Reaction conditions were 25 °C (10 min), 42 °C (60 min) and finally 72 °C (10 min).

qPCR was carried out with the QuantStudio 3 Real-Time PCR System (Applied Biosystems) and SYBR Green I (Maxima SYBR Green/ROX Kit, Thermo Scientific) in technical duplicates. Single PCR reaction mixture contained 12.5 μL of the qRT-PCR Master Mix, 2.5 μL cDNA (corresponding to 31.25 ng cDNA) and 1.5 μL of each primer (forward and reverse, 5 μM) as described in Ćosić et al.^12^. The pairs of primers used for specific amplification of *PIN-FORMED3* (*PIN3*), *ARABIDOPSIS RESPONSE REGULATOR5* (*ARR5*), *ROOT GROWTH DEFECTIVE3* (*RGD3*), the cyclin-dependent kinase gene *CDKB2;1*, and the B-type cyclin gene *CYCB2;4*, are presented in Supplementary Table S1. Specificity of presented primers corresponding to particular *A. thaliana* genes that could be found in GenBank was tested and confirmed in our previous work^12^. Potato actin gene (*PoAc58*, GenBank accession No. X55749) (Supplementary Table S1) was used as an internal control.

Conditions of qPCR reaction included initial denaturation at 95 °C for 5 min; this was followed by 40 cycles of denaturation at 95 °C for 30 s, annealing at 60 °C for 1 min, and extension at 72 °C for 1 min, with final extension at 72 °C for 10 min. Accompanying melting curve analyses were implemented by cooling the reactions to 60 °C, which was followed by increasing the temperature to 95 °C with a slope of 0.1 °C s^−1^, with continuous fluorescence measuring. The efficiency of qPCR reactions was confirmed for each analysed gene, using particular standards in 10 × serial dilutions as defined earlier in Ćosić et al.^12^. The results were analysed using QuantStudio Design and Analysis Software version 1.4 (Thermo Fisher Scientific), and presented as absolute number of transcripts per ng RNA.

### Statistical analysis

The data were analysed using SAS software (SAS Institute, 2002. SAS/STAT, ver. 9.00. SAS Institute Inc., Cary, NC, USA). To analyse the results for total endogenous CK pool as well as the content of individual CK forms distributed based on their conjugation status (CK nucleobases, CK ribosides, *O*-glucosides, *N*-glucosides and CK phosphates), analysis of variance (ANOVA) was used for each developmental stage point of each treatment, sucrose concentration being the factor analysed. The same type of analysis was used for the content analysis of *cis*Z-, *trans*Z-, DHZ- and iP-types CKs determined based on their side-chain structure, for each applied CK treatment in each developmental stage. To compare the results for mean values of particular absolute gene expression levels for T3 and T4, one-way ANOVA was employed, with sucrose concentration as analysed factor. Statistical correlation was evaluated between content of plant CK nucleobases and absolute expression of the analysed genes and presented using the Pearson’s correlation coefficient (r). The differences found among means were determined using the Fisher’s least significant difference (LSD) post-hoc test at 95% of confidence level.

## Supplementary Information

Supplementary Information

## References

[1] Mazid M, Khan TA, Mohammad F (2011). Cytokinins, a classical multifaceted hormone in plant system. J. Stress Physiol. Biochem..

[2] Wang G, Zhang G, Wu M (2016). CLE peptide signaling and crosstalk with phytohormones and environmental stimuli. Front. Plant Sci..

[3] Kieber, J.J. & Schaller, G.E. Cytokinin signaling in plant development. *Development* **145**, dev 9344 (2018). 10.1242/dev.149344 29487105

[4] Klemš *et al*. Changes in cytokinin levels and metabolism in tobacco (*Nicotiana tabacum* L.) explants during *in vitro* shoot organogenesis induced by *trans*-zeatin and dihydrozeatin. *Plant Growth Regul*. **65**, 427–437 (2011).

[5] Kamínek M, Motyka V, Vaňková R (1997). Regulation of cytokinin content in plant cells. Physiol. Plant..

[6] Ćosić T (2015). In vitro shoot organogenesis and comparative analysis of endogenous phytohormones in kohlrabi (*Brassica oleracea* var. gongylodes): effects of genotype, explant type and applied cytokinins. Plant Cell Tiss. Organ Cult..

[7] Jones B (2010). Cytokinin regulation of auxin synthesis in *Arabidopsis* involves a homeostatic feedback loop regulated via auxin and cytokinin signal transduction. Plant Cell.

[8] Cheng ZJ (2013). Pattern of auxin and cytokinin responses for shoot meristem induction results from the regulation of cytokinin biosynthesis by AUXIN RESPONSE FACTOR3. Plant Physiol..

[9] Motte H, Vereecke D, Geelen D, Werbrouck S (2014). The molecular path to *in vitro* shoot regeneration. Biotechnol. Adv..

[10] Lee DJ (2007). Genome–wide expression profiling of ARABIDOPSIS RESPONSE REGULATOR 7 (ARR7) overexpression in cytokinin response. Mol. Genet. Genomics.

[11] Rashotte AM, Carson SDB, To JPC, Kieber JJ (2003). Expression profiling of cytokinin action in Arabidopsis. Plant Physiol..

[12] Ćosić (2019). Expression profiles of organogenesis-related genes over the time course of one-step *de novo* shoot organogenesis from intact seedlings of kohlrabi. J. Plant Physiol..

[13] Tamaki H (2009). Identification of novel meristem factors involved in shoot regeneration through the analysis of temperature-sensitive mutants of Arabidopsis. Plant J..

[14] Riou-Khamlichi C, Huntley R, Jacqmard A, Murray JAH (1999). Cytokinin activation of Arabidopsis cell division through a D-type cyclin. Science.

[15] Rosa YBCJ, Aizza LCB, Armanhi JSL, Dornelas MC (2013). A *Passiflora* homolog of a D–type cyclin gene is differentially expressed in response to sucrose, auxin, and cytokinin. Plant Cell Tiss. Organ Cult..

[16] Planchais S (1997). Roscovitine, a novel cyclin–dependent kinase inhibitor, characterizes restriction point and G2/M transition in tobacco BY–2 cell suspension. Plant J..

[17] Laureys F (1998). Zeatin is indispensable for the G2–M transition in tobacco BY–2 cells. FEBS Lett..

[18] Zhang K, Diederich L, John PCL (2005). The cytokinin requirement for cell division in cultured *Nicotiana plumbaginifolia* cells can be satisfied by yeast Cdc25 protein tyrosine phosphatase. Implications for mechanisms of cytokinin response and plant development. Plant Physiol..

[19] León P, Sheen J (2003). Sugar and hormone connections. Trends Plant Sci..

[20] Gibson SI (2004). Sugar and phytohormone response pathways: navigating a signalling network. J. Exp. Bot..

[21] Skylar A, Sung F, Hong F, Chory J, Wu X (2011). Metabolic sugar signal promotes *Arabidopsis* meristematic proliferation via G2. Dev. Biol..

[22] Koch KE (1996). Carbohydrate–modulated gene expression in plants. Annu. Rev. Plant Physiol. Plant Mol. Biol..

[23] Kushwah S, Laxmi A (2017). The interaction between glucose and cytokinin signaling in controlling *Arabidopsis thaliana* seedling root growth and development. Plant Signal. Behav..

[24] Sakr S (2018). The sugar-signaling hub: Overview of regulators and interaction with the hormonal and metabolic network. Int. J. Mol. Sci..

[25] Smeekens S, Rook F (1997). Sugar sensing and sugar-mediated signal transduction in plants. Plant Physiol..

[26] Jefferson R, Goldsbrough A, Bevan M (1990). Transcriptional regulation of a patatin–1 gene in potato. Plant Mol. Biol..

[27] Yokoyama R (1994). The rolC promoter of *Agrobacterium rhizogenes* Ri plasmid is activated by sucrose in transgenic tobacco plants. Mol. Gen. Genet..

[28] Lu C-A, Ho T-D, Ho S-L, Yu S-M (2002). Three novel MYB proteins with one DNA binding repeat mediate sugar and hormone regulation of a-amylase gene expression. Plant Cell.

[29] Cheng W-H (2002). A unique short-chain dehydrogenase/ reductase in Arabidopsis abscisic acid biosynthesis and glucose signaling. Plant Cell.

[30] Riou-Khamlichi C, Menges M, Healy JMS, Murray JAH (2000). Sugar control of the plant cell cycle: Differential regulation of Arabidopsis D–type cyclin gene expression. Mol. Cell. Biol..

[31] Sami F, Yusuf M, Faizan M, Faraz A, Hayat S (2016). Role of sugars under abiotic stress. Plant Physiol. Biochem..

[32] Moore B (2003). Role of the *Arabidopsis* glucose sensor HXK1 in nutrient, light, and hormonal signaling. Science.

[33] Hartig K, Beck E (2006). Crosstalk between auxin, cytokinins, and sugars in the plant cell cycle. Plant Biol..

[34] Lee S-T, Huang W-L (2013). Cytokinin, auxin, and abscisic acid affects sucrose metabolism conduce to de novo shoot organogenesis in rice (*Oryza sativa* L.) callus. Bot. Stu..

[35] Ikeda Y, Koizumi N, Kusano T, Sano H (1999). Sucrose and cytokinin modulation of WPK4, a gene encoding a SNF1-related protein kinase from wheat. Plant Physiol..

[36] Kushwah S, Laxmi A (2014). The interaction between glucose and cytokinin signal transduction pathway in *Arabidopsis thaliana*. Plant Cell Environ..

[37] Barbier F (2015). Sucrose is an early modulator of the key hormonal mechanisms controlling bud outgrowth in *Rosa hybrida*. J. Exp. Bot..

[38] Ćosić (2013). In vitro plant regeneration from immature zygotic embryos and repetitive somatic embryogenesis in kohlrabi (*Brassica oleracea* var. *gongylodes*). Vitro Cell. Dev. Biol. Plant.

[39] Ćosić T (2020). Effects of different types of sugars and plant growth regulators on kohlrabi seedling growth and development *in vitro*. Arch. Biol. Sci..

[40] Kamínek M (2000). Purine cytokinins: a proposal of abbreviations. Plant Growth Regul..

[41] Aremu AO (2014). How does exogenously applied cytokinin type affect growth and endogenous cytokinins in micropropagated *Merwilla plumbea*?. Plant Cell Tiss. Organ Cult..

[42] Gibson SI (2005). Control of plant development and gene expression by sugar signaling. Curr. Opin. Plant Biol..

[43] Yaseen M, Ahmad T, Sablok G, Standardi A, Hafiz IA (2013). Review: role of carbon sources for *in vitro* plant growth and development. Mol. Biol. Rep..

[44] Borisjuk L (2003). Energy status and its control on embryogenesis of legumes: ATP distribution within *Vicia faba* embryos is developmentally regulated and correlated with photosynthetic capacity. Plant J..

[45] Desjardins, Y., Hdider, C. & de Riek, J. Carbon nutrition *in vitro* - regulation and manipulation of carbon assimilation in micropropagated systems in *Automation and Environmental Control in Plant Tissue Culture* (eds. Aitken–Christie, J., Kozai, T. & Smith, M.L.) 441– 471 (Springer Nature, Switzerland, 1995).

[46] Gabryszewska E (2010). The effects of glucose and growth regulators on the organogenesis of *Paeonia lactiflora* Pall. in vitro. J. Fruit Ornam. Plant Res..

[47] Dekkers, B.J.W. & Smeekens, S. Sugar and abscisic acid regulation of germination and transition to seedling growth in *Seed development, dormancy and germination* (eds. Bradford, K. & Nonogaki, H.) 305–327 (Blackwell Publishing, Oxford, UK, 2007).

[48] Zhou L, Jang JC, Jones TL, Sheen J (1998). Glucose and ethylene signal transduction crosstalk revealed by an Arabidopsis glucose-insensitive mutant. PNAS.

[49] Gibson SI, Laby RJ, Kim D (2001). The *sugar-insensitive1* (*sis1*) mutant of Arabidopsis is allelic to *ctr1*. Biochem. Biophys. Res. Commun..

[50] Lu B (2020). Overexpression of melon tonoplast sugar transporter CmTST1 improved root growth under high sugar content. Int. J. Mol. Sci..

[51] George EF (1993). Plant propagation by tissue culture in *Part 1: the technology*, 1–574.

[52] Wang H-L, Lee P-D, Liu L-F, Su J-C (1999). Effect of sorbitol induced osmotic stress on the changes of carbohydrate and free amino acid pools in sweet potato cell suspension cultures. Bot. Bull. Acad. Sinica.

[53] Biahoua A, Bonneau L (1999). Control of in vitro somatic embryogenesis of the spindle tree (*Euonymus europaeus* L.) by the sugar type and the osmotic potential of the culture medium. Plant Cell Rep..

[54] Lapenã L, Pérez-Bermúdez P, Segura J (1988). Morphogenesis in hypocotyl cultures of *Digitalis obscura*: influence of carbohydrate levels and sources. Plant Sci..

[55] Rognoni S, Teng S, Arru L, Smeekens SC, Perata P (2007). Sugar effect on early seedling development in Arabidopsis. Plant Growth Regul..

[56] Guo B, Abbasi BH, Zeb A, Xu LL, Wei YH (2011). Thidiazuron: a multi-dimensional plant growth regulator. Afr. J. Biotechnol..

[57] Dewir YH, Nurmansyah NY, Teixeira da Silva JA (2018). Thidiazuron-induced abnormalities in plant tissue cultures. Plant Cell Rep..

[58] Zhang CG, Li W, Mao YF, Zhao DL, Dong W, Guo GQ (2005). Endogenous hormonal levels in *Scutellaria baicalensis* calli induced by thidiazuron. Russ. J. Plant Physiol..

[59] Shirani S, Mahdavi F, Maziah M (2010). Morphological abnormality among regenerated shoots of banana and plantain (*Musa* spp.) after in vitro multiplication with TDZ and BAP from excised shoot-tips. Afr. J. Biotechnol..

[60] Wang SY, Jiao HJ, Faust M (1991). Changes in metabolic enzyme activities during TDZ-induced bud break of apple. Hort. Sci..

[61] Cuesta C (2012). Endogenous cytokinin profiles and their relationships to between-family differences during adventitious caulogenesis in *Pinus pinea* cotyledons. J. Plant Physiol..

[62] Montalbán IA, Novák O, Rolčik J, Strnad M, Moncaleán P (2013). Endogenous cytokinin and auxin profiles during *in vitro* organogenesis from vegetative buds of *Pinus radiata* adult trees. Physiol. Plant..

[63] Raspor M (2012). Cytokinin profiles of *AtCKX2*-overexpressing potato plants and the impact of altered cytokinin homeostasis on tuberization *in vitro*. J. Plant Growth Regul..

[64] Gujjar RS, Supaibulwatana K (2019). The mode of cytokinin functions assisting plant adaptations to osmotic stresses. Plants.

[65] Arrom L, Munné-Bosch S (2012). Hormonal changes during flower development in floral tissues of Lilium. Planta.

[66] Sairanen I (2012). Soluble carbohydrates regulate auxin biosynthesis via PIF proteins in Arabidopsis. Plant Cell.

[67] Gordon SP (2007). Pattern formation during *de novo* assembly of the *Arabidopsis* shoot meristem. Development.

[68] Huang WL, Lee CH, Chen YR (2012). Levels of endogenous abscisic acid and indole-3-acetic acid influence shoot organogenesis in callus cultures of rice subjected to osmotic stress. Plant Cell Tiss. Organ Cult..

[69] Takei K, Sakakibara H, Sugiyama T (2001). Identification of genes encoding adenylate isopentenyltransferase, a cytokinin biosynthesis enzyme, *Arabidopsis thaliana*. J. Biol. Chem..

[70] Vanková R., Gaudinová A., Kamínek, M. & Eder, J. The effect of interaction of synthetic cytokinin and auxin on production of natural cytokinins by immobilized tobacco cells in *Physiology and Biochemistry of Cytokinins in Plants* (eds. Kamínek, M., Mok, D.W.S. & Zazímalová, E.) 47–51 (SPB Academic Publishing, The Hague, The Netherlands, 1992).

[71] Nisler J (2016). Novel thidiazuron-derived inhibitors of cytokinin oxidase/dehydrogenase. Plant Mol. Biol..

[72] Romanov GA, Lomin SN, Schmülling T (2018). Cytokinin signaling: from the ER or from the PM? That is the question!. New Phytol..

[73] Murch SJ, Victor JMR, Krishnaraj S, Saxena PK (1999). The role of proline in thidiazuron-induced somatic embryogenesis of peanut. Vitro Cell Dev. Biol..

[74] Trewavas, A.J. The importance of individuality in *Plant responses to environmental stresses* (ed. Lerner, H.R.) 27–43 (Marcel Dekker, New York, 1999).

[75] Kiba T, Takebayashi Y, Kojima M, Hitoshi Sakakibara H (2019). Sugar-induced *de novo* cytokinin biosynthesis contributes to Arabidopsis growth under elevated CO_2_. Sci. Rep..

[76] Stokes ME, Chattopadhyay A, Wilkins O, Nambara E, Campbell MM (2013). Interplay between sucrose and folate modulates auxin signaling in Arabidopsis. Plant Physiol..

[77] Takei K (2004). AtIPT3 is a key determinant of nitrate-dependent cytokinin biosynthesis in Arabidopsis. Plant Cell Physiol..

[78] Ohkama N (2002). Regulation of sulfur–responsive gene expression by exogenously applied cytokinins in *Arabidopsis thaliana*. Plant Cell Physiol..

[79] Woo J (2012). The response and recovery of the *Arabidopsis thaliana* transcriptome to phosphate starvation. BMC Plant Biol..

[80] Kushwah S, Jones AM, Laxmi A (2011). Cytokinin interplay with ethylene, auxin and glucose signaling controls Arabidopsis seedling root directional growth. Plant Physiol..

[81] Hwang I, Sheen J (2001). Two-component circuitry in Arabidopsis cytokinin signal transduction. Nature.

[82] Cary AJ, Che P, Howell SH (2002). Developmental events and shoot apical meristem gene expression patterns during shoot development in *Arabidopsis thaliana*. Plant J..

[83] Leibfried A (2005). WUSCHEL controls meristem function by direct regulation of cytokinin-inducible response regulators. Nature.

[84] Murashige T, Skoog F (1962). A revised medium for rapid growth and bio assays with tobacco tissue cultures. Physiol. Plant..

[85] Linsmaier EM, Skoog F (1965). Organic growth factor requirements of tobacco tissue cultures. Physiol. Plant..

[86] Dobrev PI, Vankova R (2012). Quantification of abscisic acid, cytokinin, and auxin content in salt-stressed plant tissues. Methods Mol. Biol..

[87] Djilianov DL (2013). Dynamics of endogenous phytohormones during desiccation and recovery of the resurrection plant species *Haberlea rhodopensis*. J. Plant Growth Regul..

[88] Gasic K, Hernandez A, Korban SS (2004). RNA extraction from different apple tissues rich in polyphenols and polysaccharides for cDNA library construction. Plant Mol. Biol. Rep..

