# Supplementary material for: Sucrose interferes with endogenous cytokinin homeostasis and expression of organogenesis-related genes during de novo shoot organogenesis in kohlrabi
Source: Sci Rep. 2021 Mar 22;11:6494. doi: 10.1038/s41598-021-85932-w (PMC7985405; doi:10.1038/s41598-021-85932-w)
Supplement: Supplementary file 1 — Supplementary Information [file 41598_2021_85932_MOESM1_ESM.pdf]

Sucrose interferes with endogenous cytokinin homeostasis and expression of organogenesis-related genes during *de novo* shoot organogenesis in kohlrabi

Tatjana Ćosić, Václav Motyka, Jelena Savić, Martin Raspor, Marija Marković, Petre I. Dobrev, Slavica Ninković

Supplementary Information

Supplementary Table S1. Sequences of specific gene forward (F) and reverse (R) primers used in qPCR analysis.

Gene	GenBank™ access number	Primer sequence		Expected amplicon length
		Forward (5’- 3’)	Reverse (5’ - 3’)	
PIN3	NM_105762.2	TAAACCAGCGTGATCGGAAG	ATTGTGCCCTTTGTGTTTGC	104 bp
ARR5	NM_114679	TCTATGCCTGGGATGACTGGA	TCACATCAGCTAATTTCACAGGC	175 bp
RGD3	NM_115288	GACGTGGAGTCATCTAGGCA	AGCCATCAGCGGCAAGATTA	162 bp
CDKB2;1	NM_106304	GTACGAGCCAGCGAAACGAA	GCAGCACACTAGAGATATGCTTG	189 bp
CYCB2;4	NM_106281	GCATCGGCGATCTACACTGC	CCCTGCCTTGTGATGCAAAC	141 bp
PoAc58	X55749	TGTTGGACTCTGGTGATGGTG	AGTAACCACGCTCAGTGAGGA	141 bp
